# COVID-19 Vaccination in a Former Fukushima Nuclear Accident Evacuation Area: COVID-19 Vaccination for Former Evacuees

**DOI:** 10.1017/dmp.2022.291

**Published:** 2022-12-12

**Authors:** Naomi Ito, Sachiko Yoshida, Mika Sato, Kiyotaka Yasui, Yuki Sonoda, Masaharu Tsubokura

**Affiliations:** 1 Department of Radiation Health Management, Fukushima Medical University School of Medicine, Fukushima, Japan; 2 School of Public Health, Teikyo University, Itabashi-ku, Tokyo, Japan; 3 Department of Health Nursing of International Radiation Exposure, Fukushima Medical University School of Medicine, Fukushima, Japan; 4 Takebayashi Sadakichi Memorial Clinic, Tokiwa Foundation, Iwaki, Fukushima, Japan

**Keywords:** COVID-19 vaccination, evacuation area, Fukushima disaster, residents’ health, access to health service

## Abstract

After the Fukushima accident in 2011, approximately 160000 residents were forced to evacuate the site. The evacuation order has since been lifted and the Japanese government is now advancing a return strategy. As the return proceeds, deterioration of health indicators and measures in the municipalities around the nuclear power plant remains unchanged. This affected the local governments’ coronavirus disease (COVID-19) vaccination drive during the COVID-19 pandemic. In Japan, municipalities keep track of residents’ information and implement health-related measures. However, many residents evacuated the town, thus leaving their registered residence. With long-term evacuation and few returnees, it was difficult for government officials to constantly monitor the residents’ health. Therefore, there is an urgent need to maintain residents’ records and ensure that they receive health services without any gap. This report aimed to provide implications for post-disaster community health services and support as observed during the COVID-19 vaccination program at a disaster site.

## Introduction

Negative effects of the Fukushima accident on the residents’ health were diverse because they were influenced not only by the radiation exposure but also by lifestyle changes and social environment. These effects were revealed over time in different situations during the restoration process.^1^

Health indicators have been reported to deteriorate for evacuated residents because life at the evacuation destination is accompanied by major changes in social environment.^2,3^ Murakami *et al*. reported that the depression experienced by long-term evacuees was very severe,^4^ while Iwasaki *et al*. mentioned that strengthening social capital can help improve the resilience of affected residents.^5^ Kobashi *et al*., reported that evacuees used an extremely high level of care services.^6^ Although various health problems were noted, people continued to evacuate. The resultant behavior of municipalities and the kind of services required in such situations, however, remain unclear.

In Japan, resident registration is processed by the municipalities that also undertake health-related measures. Similarly, municipalities are responsible for coronavirus disease (COVID-19) vaccination for all registered residents, including the ones who have been evacuated from the municipalities. This system is like the health follow-up carried out after the Fukushima accident. Delineating the difficulties experienced during the vaccination program may be useful in understanding the challenges of implementing health measures for the Fukushima accident evacuees.

**Figure 1.**
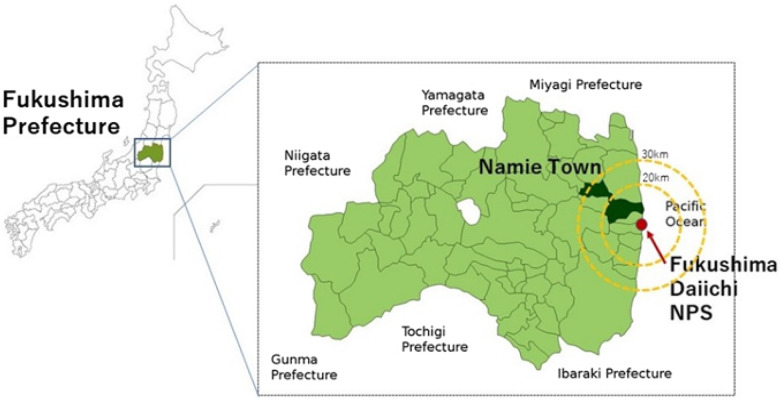
Location of Namie Town, Fukushima Prefecture, and distance from the Fukushima Daiichi nuclear power plant.

Namie Town, with a population of about 21000, located 4 - 30 km from the Fukushima Daiichi nuclear power plant, was designated as an evacuation area (Figure 1). On April 1, 2019, the evacuation order was lifted, and residents gradually began to return. Currently, the returned population is 1248, which is 6.3% of the town’s registered population (19869) as of November 30, 2021.^7^ As many residents continue to remain in their evacuated locations, it is vital to collect their health follow-up information. Municipalities have been taking various measures for the COVID-19 pandemic and vaccination^8,9^; however, COVID-19 has reportedly become a major health issue in evacuation areas after the Fukushima accident.

This study aimed to report the issues faced and measures taken to solve the health problems of residents who had been evacuated for a long time since the Fukushima accident, and to evaluate the issues encountered in performing vaccinations for the returning evacuees.

## Narrative

Vaccination in Namie Town commenced in May 2021, and by the end of November 1724 residents (84.6%) had completed the second vaccination dose. Those residents who evacuated the town were vaccinated at the evacuation site, and the second vaccination rate for all residents, including evacuees, was 80.3%.

The vaccination team was set up in the public office and managed the work with a few staff members. Under the local government-led vaccine program policy, the town sent out a notice to all the registered residents. While 90% of the registered residents still lived outside the evacuated town (evacuated residents), the vaccination target was limited to the residents living in the town (returned residents) according to the national policy. Despite this, the vaccination team sent out vaccination tickets, not only to the residents in the town (returned residents), but also to those outside the town (evacuated residents), and enclosed ID numbers and guidebooks according to the progress and rules of each destination municipality. Some evacuees had to take the vaccination by themselves, and some experienced vaccination delays. Municipalities essentially provide health support to registered residents but are not accustomed to providing constant health support to evacuees. For a smooth vaccination drive of Namie’s registered residents at their evacuation sites, the Namie public office staff often had to correspond with the officials of the residents’ evacuation sites to bridge the gap.

Many health care workers were needed to carry out the vaccination within a short period. Help from outside was sought because of the insufficiency of human resources in the town.^10^ The only municipal clinic in town was busy with its routine medical work and was consequently unable to work with the public office. The support of the Fukushima Medical University staff, along with the cooperation of the authors of this paper, was of great help but was still insufficient. In 2022, the booster vaccination drive started. The drive, along with the added vaccine request survey as there were 2 types of vaccines, began ahead of schedule. This further increased the burden and caused exhaustion in public officials. Additionally, the commencement of vaccination drive for children added to the workload.

The supply of inoculation equipment is as important as human resources. The vaccination team balanced the resources and vaccine supplies. For example, freezers were distributed to the disaster area without discrimination. Moreover, consumables (gloves and gowns) were available for all the registered residents, but only those who returned had received vaccination, thus leading to overstocking.

The vaccination records of all the registered residents at each vaccination site nationwide remains incomplete. Many personnel were involved, and many data entry errors were noted, making it difficult to share this information. Verification and data entry of vaccination records from other local governments and medical institutions is in progress. For example, for each discrepancy in dose interval, we first contact the relevant institution to trace the cause, then establish measures to prevent recurrence, and finally write an incident report. This is necessary because knowing the correct date of the second vaccination is important in determining the date of the third dose. However, this approach was not taken for those with disabilities or the older population. Furthermore, we did not ascertain the number of people with disabilities in the town that were vaccinated.

## Discussion

This report revealed that providing healthcare services to evacuees was a major challenge in the Fukushima disaster area. The shortage of medical institutions and staff has always been an issue; however, this problem was amplified after the earthquake because several residents did not return even after the evacuation order was lifted.^11^ To carry out vaccinations in a short period of time while prioritizing older individuals, enough medical staff and local government officials is required. Therefore, future vaccination drives initiated by the local government should secure adequate medical system and human resource.

It appears difficult to contact and supply health support to long-term evacuees after the Fukushima accident. Due to the prolonged evacuation, the national government stipulated that evacuees could receive similar health services at evacuation destinations.^12^ However, this report found that the local governments tend to vaccinate their residents first and then evacuees. In addition, older people should be prioritized in the vaccination program. Therefore, long-term evacuation strategies and attribution of residence should be reevaluated. It is important for individuals to receive the necessary medical support, regardless of their place of residence. However, it is challenging to understand the situation of the residents in scattered evacuation destinations and provide health support services with the limited number of local government officials. Support work that was challenging to manage by the local governments with the help of experts from other organizations includes not only vaccination, but also medical examinations and follow-up. This kind of situation is unacceptable 10 years after the Fukushima accident, wherein 90% of the Namie evacuated residents did not return home.

It is time to reconsider the attribution of the residence of long-term evacuees since the Fukushima accident. After 6 years of evacuation of Namie Town, residents are gradually returning. Although 10 years have passed since the accident, more than 90% of the residents still live outside the town. During the period of the evacuation order, people cherished their unity with the Namie townspeople,^13^ no matter where they were. However, now that the evacuation order has been lifted and the residents are returning, their mindsets are changing. It is also necessary to improve the municipality system to disseminate information about the services available in the municipalities where evacuated residents live.

This report provides suggestions for future health care services and community support in post-disaster areas. With the ongoing vaccination drive, it is necessary to consider how to improve the medical system, prioritize groups of residents, and provide effective services to all.

## Abbreviation

COVID-19, Coronavirus Disease-2019
